# Automated Segmentation of Midbrain Structures in High-Resolution Susceptibility Maps Based on Convolutional Neural Network and Transfer Learning

**DOI:** 10.3389/fnins.2022.801618

**Published:** 2022-02-10

**Authors:** Weiwei Zhao, Yida Wang, Fangfang Zhou, Gaiying Li, Zhichao Wang, Haodong Zhong, Yang Song, Kelly M. Gillen, Yi Wang, Guang Yang, Jianqi Li

**Affiliations:** ^1^Shanghai Key Laboratory of Magnetic Resonance, School of Physics and Electronic Science, East China Normal University, Shanghai, China; ^2^Department of Radiology, Weill Medical College of Cornell University, New York, NY, United States

**Keywords:** midbrain structure, automated segmentation, high-resolution quantitative susceptibility mapping, convolutional neural network, transfer learning

## Abstract

**Background:**

Accurate delineation of the midbrain nuclei, the red nucleus (RN), substantia nigra (SN) and subthalamic nucleus (STN), is important in neuroimaging studies of neurodegenerative and other diseases. This study aims to segment midbrain structures in high-resolution susceptibility maps using a method based on a convolutional neural network (CNN).

**Methods:**

The susceptibility maps of 75 subjects were acquired with a voxel size of 0.83 × 0.83 × 0.80 mm^3^ on a 3T MRI system to distinguish the RN, SN, and STN. A deeply supervised attention U-net was pre-trained with a dataset of 100 subjects containing susceptibility maps with a voxel size of 0.63 × 0.63 × 2.00 mm^3^ to provide initial weights for the target network. Five-fold cross-validation over the training cohort was used for all the models’ training and selection. The same test cohort was used for the final evaluation of all the models. Dice coefficients were used to assess spatial overlap agreement between manual delineations (ground truth) and automated segmentation. Volume and magnetic susceptibility values in the nuclei extracted with automated CNN delineation were compared to those extracted by manual tracing. Consistencies of volume and magnetic susceptibility values by different extraction strategies were assessed by Pearson correlation coefficients and Bland-Altman analyses.

**Results:**

The automated CNN segmentation method achieved mean Dice scores of 0.903, 0.864, and 0.777 for the RN, SN, and STN, respectively. There were no significant differences between the achieved Dice scores and the inter-rater Dice scores (*p* > 0.05 for each nucleus). The overall volume and magnetic susceptibility values of the nuclei extracted by the automatic CNN method were significantly correlated with those by manual delineation (*p* < 0.01).

**Conclusion:**

Midbrain structures can be precisely segmented in high-resolution susceptibility maps using a CNN-based method.

## Introduction

The red nucleus (RN), substantia nigra (SN), and subthalamic nucleus (STN) are small ganglia located in the midbrain and of great importance in regulating motor control, cognition, and emotion (Boecker et al., 2008). Accurate segmentation in these structures is important for analyzing structural variations and iron concentration changes. Evaluation of morphological degeneration and iron deposition can aid clinicians in early detection and diagnosis of neurodegenerative diseases, including Alzheimer’s disease (AD), Parkinson’s disease (PD), and multiple sclerosis (MS) (Wang et al., 2017). In addition, precise delineation of the STN can provide effective clinical treatment assistance for PD patients requiring deep brain stimulation (DBS) surgery (Dimov et al., 2019).

Midbrain nuclei are typically manually segmented, which is extremely time-consuming and dependent on evaluator experience. Automated segmentation methods are advantageous for faster and more reproducible results. Current automated brain segmentation methods were developed using automated brain mapping from brain atlases, where the most commonly used atlases were based on T1 contrast (Lancaster et al., 2000). However, automatic segmentation using these atlases is challenging in deep gray matter nuclei of the midbrain due to its low T1 contrast (Xiao et al., 2014). Although the RN, SN and STN appear to be moderately hypointense on T2-weighted (T2w) images due to iron deposition, clinical T2w images on 3T or 1.5T systems do not differentiate the STN from the adjacent SN (Xiao et al., 2014). Direct visualization of the STN may require ultrahigh-field-strength scanners, such as 7T (Schafer et al., 2012; Kim et al., 2019), which are not widely used in clinical practice.

Quantitative susceptibility mapping (QSM) can obtain *in vivo* tissue magnetic susceptibility distribution by using gradient echo phase images (de Rochefort et al., 2010). QSM provides an excellent contrast in deep gray matter regions because these structures are characterized by their high paramagnetic iron content, and are therefore clearly visible and easily distinguishable in susceptibility maps (Liu et al., 2010, 2013a; Haacke et al., 2015; Wang and Liu, 2015). The susceptibility maps yield a superior contrast-to-noise ratio in the depiction of the STN when compared with T2w images, and the true ellipsoidal shape of the STN is reliably reflected, which permits its distinction from the SN (Liu et al., 2013a). Combining magnetic susceptibility and T1 contrast in a multi-atlas approach yielded improved accuracy and reliability for automated segmentation because it can potentially model a greater amount of anatomical variability (Garzon et al., 2018; Li et al., 2019). However, existing multi-atlas approaches based on QSM cannot discriminate the STN from the SN, due to the low spatial resolution of the acquired images, or challenges in multiple registrations when aligning atlases to the target images.

In recent years, convolutional neural network (CNN) has been successfully applied to the segmentation of brain tissue, tumor and MS lesions (Brosch et al., 2016; Havaei et al., 2017). CNN has also been used in segmenting sub-cortical brain structures in traditional T1-weighted MRI (Dolz et al., 2018). Deep learning approaches achieved better overall performance in automated segmentation of subcortical brain structures, compared with atlas-based approaches and algorithmic approaches (Pagnozzi et al., 2019; Beliveau et al., 2021). To overcome the issue that CNN-based methods require large training datasets, the transfer learning can be applied, where a network pre-trained with a much larger dataset is used to initialize the target network weights, significantly reducing the demand of training data and training time for the target network (Pan and Yang, 2009; Xu et al., 2017). Transfer learning has been used in medical image segmentation and achieved good results in segmenting brain tissue (Ataloglou et al., 2019). To our knowledge, no study explored the potential of the CNN model for segmenting midbrain structures in susceptibility maps.

The purpose of this study was to segment three midbrain nuclei, the RN, SN, and STN, by taking advantage of the high-resolution susceptibility maps and CNN. The high-resolution susceptibility maps were acquired with a nearly isotropic voxel size of 0.83 × 0.83 × 0.80 mm^3^ for precise characterization of the midbrain nuclei. A deeply supervised attention U-net with the transfer learning algorithm was applied to obtain the segmentation results. The Dice coefficients between the results with the automated segmentation method and the manual ground truth were calculated to evaluate the performance of automatic segmentation. Furthermore, the volume and magnetic susceptibility values in the nuclei extracted with automated CNN delineation were also compared with those obtained by manual tracing.

## Materials and Methods

### Datasets

Quantitative susceptibility mapping from 100 subjects (53 males and 47 females; mean age = 43.7 ± 15.6 years) with a voxel size of 0.63 × 0.63 × 2.0 mm^3^ from a previous study (Li et al., 2018) were used as the source dataset to pre-train the network, and QSM data with a voxel size of 0.83 × 0.83 × 0.80 mm^3^ from a new cohort of 75 subjects (40 male and 35 female; mean age = 33.7 ± 13.4 years) were used as the target dataset. This study was approved by the local institutional review board and written informed consents were obtained from all participants.

All participants in the source dataset were scanned on a clinical 3T MR imaging system (Trio Tim, Siemens Healthcare, Erlangen, Germany) equipped with a 12-channel head matrix coil. Susceptibility maps were generated from the 3D spoiled unipolar-readout multi-echo GRE sequence acquired in the axial plane with the following imaging parameters: repetition time (TR) = 60 ms, first echo time (TE_1_) = 6.8 ms, echo spacing (ΔTE) = 6.8 ms, number of echoes = 8, flip angle = 15°, field of view (FOV) = 240 × 180 mm^2^, matrix size = 384 × 288, slice thickness = 2 mm, number of slices = 96, voxel size = 0.63 × 0.63 × 2.00 mm^3^, scan time = 7 min 52 s. A generalized auto-calibrating partially parallel acquisition (GRAPPA) with an acceleration factor of 2 in the right-left direction and elliptical sampling were used to reduce acquisition time.

All subjects in the target dataset were scanned on another 3T MRI scanner (Prisma Fit, Siemens Healthcare, Erlangen, Germany) equipped with a 20-channel head coil. Susceptibility maps were generated from a 3D spoiled bipolar-readout multi-echo GRE sequence with the following parameters: TR = 31 ms, TE_1_ = 4.07 ms, ΔTE = 4.35 ms, number of echoes = 6, flip angle = 12°, FOV = 240 × 200 mm^2^, matrix size = 288 × 240, slice thickness = 0.8 mm, number of slices = 192, parallel imaging acceleration factor = 2, voxel size = 0.83 × 0.83 × 0.80 mm^3^, scan time = 7 min 22 s. Images were acquired in the oblique-axial plane parallel to the anterior commissure–posterior commissure line (AC–PC line).

During scanning, foam pads were placed around each subject’s head to minimize head motion.

### Quantitative Susceptibility Mapping Reconstruction

Susceptibility maps were reconstructed using the Morphology Enabled Dipole Inversion (MEDI) toolbox^1^. A brain extraction tool (BET) was first used to segment the brain tissue from the magnitude images. Then, the phase shift of even echoes induced by the gradient delay and eddy current was estimated and corrected for the data in the target domain (Li et al., 2015). The field map was estimated by performing a one-dimensional temporal unwrapping of the phase on each voxel followed by a nonlinear least-squares fit of the temporally unwrapped phases in each voxel over TE (Liu et al., 2013b). To address frequency aliasing on the field map, a Laplacian-based unwrapping algorithm was applied (Schofield and Zhu, 2003). The tissue field was separated from the background field by applying a projection onto dipole fields procedure (PDF) (Liu et al., 2011). Susceptibility maps were calculated by MEDI with automatic uniform cerebrospinal fluid zero reference (MEDI + 0) (Liu et al., 2018).

### Manual Tracing

**FIGURE 1:**
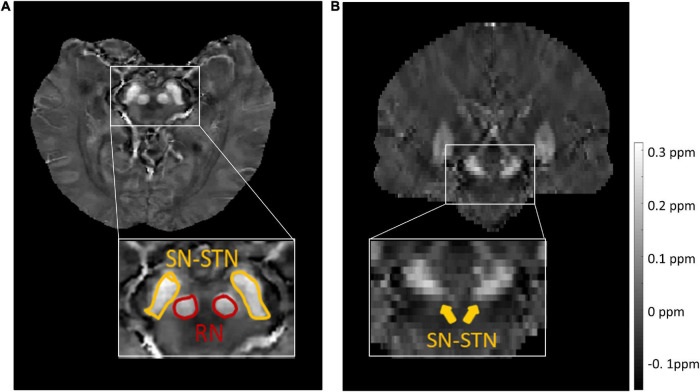
Representative images and labels of midbrain structures in axial **(A)** and coronal **(B)** susceptibility maps of a female subject (46 y/o) from the source dataset with a voxel size of 0.63 × 0.63 × 2.00 mm^3^. Yellow arrows in panel **(B)** mark the regions covering the SN and STN. RN, red nucleus; SN, substantia nigra; STN, subthalamic nucleus.

**FIGURE 2:**
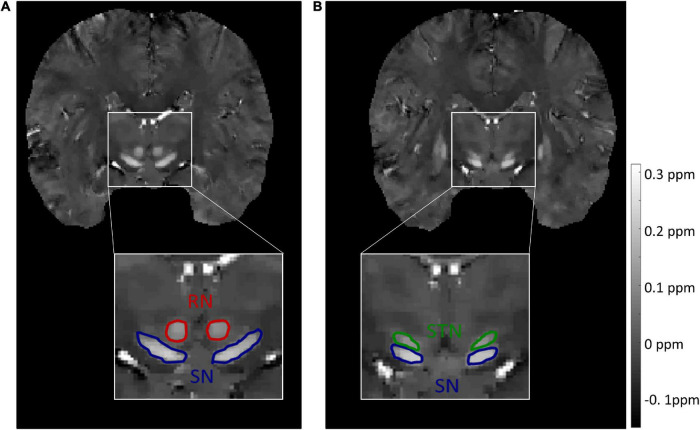
Representative images and labels of midbrain structures in coronal susceptibility maps of a subject (male, 24 y/o) from the target dataset with a voxel size of 0.83 × 0.83 × 0.80 mm^3^. **(A)** The borders of the RN and SN. **(B)** The borders of the SN and STN. RN, red nucleus; SN, substantia nigra; STN, subthalamic nucleus.

For the source dataset, the SN and STN were delineated as a whole in the axial view (Figure 1A), because there was no clear boundary between the SN and STN, even in the coronal view (Figure 1B). But for the target dataset with an approximate isotropic voxel size of 0.83 × 0.83 × 0.80 mm^3^, the true ellipsoidal shape of the STN was reliably distinguished from the SN in the coronal view (Figure 2B). Therefore, the RN, SN, and STN were delineated in the coronal view (Figure 2). A rater (6 years of neuroimaging experience), who was blinded to subject demographics, manually drew regions of interest (ROIs) on the susceptibility maps. Another rater (4 years of neuroimaging experience) manually drew the ROIs in the test cohort of the target domain. ROIs covered each bilateral structure on all sections where the deep nuclei were visible. Manual tracing was performed using ITK-SNAP software^2^.

### Data Preprocessing

All slices including the ROIs and adjacent slices without ROIs were selected along the slice direction as the input data of the network. We used a CNN model to segment the midbrain nuclei regions and the 5-fold cross-validation over the training cohort was used for model training and selection. Three consecutive 2D images were used as input and the ground truth was corresponding to the middle slice image. Transverse slices in the source dataset were center-cropped to 128 × 128 before being used as input. For the target domain images, coronal slices center-cropped to 96 × 96 were used. Statistics on midbrain deep nuclei size in both the source and target datasets were used to ensure that no nuclei region was lost due to cropping.

To increase the robustness of the model, an on-line data augmentation strategy (Shan, 2019) was used all through the training process, which applied random shifting within ±15 pixels, random rotating within ±10°, and random shearing from 0.8 to 1.2 to each sample used in each epoch. In the source dataset, 64 subjects were used for model training and the number of slices for each subject was 96, so the total number of augmented training images was about 5.5 million. In the target dataset, 60 subjects were used for model training and the number of slices for each subject was 192, so the number of training images after the data augmentation was about 5.2 million (Detailed description on the estimation method is provided in the Supplementary Material).

### Convolutional Neural Network Architecture

**FIGURE 3:**
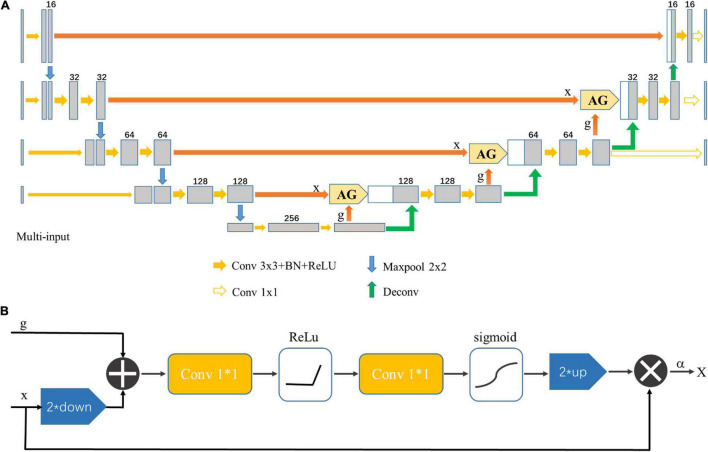
**(A)** Multi-input Attention U-net network structure. AG indicates that the attention mechanism was applied. **(B)** Schematic diagram of the AG. The input feature (x) was scaled by the attention coefficient (α) calculated in AG, in which the target area was selected by analyzing the contextual information provided by the activation function and the gate signal (g) obtained from a lower scale.

The multi-input Attention U-net model was used to segment the midbrain nucleus, which is shown in Figure 3A. The standard U-net contains an encoder and a decoder part, and each part consists of four downsampling and upsampling stages. The attention gates (AGs) were incorporated into the U-net to highlight salient features that were passed through the skip connections (Oktay et al., 2018). The architecture of the proposed AG is shown in Figure 3B. The coarse level and fine level features were fed into the AGs to get the attention map so the scaled features could be specific to local information. Input features (*x*) were fed into a convolutional layer with a kernel size of 1 × 1 and stride of 2 to obtain features with the same size as the gate signal (*g*) in the decoder part and then added with *g*. The attention coefficient (α) was obtained after features went through two 1 × 1 convolutional layers, ReLU, sigmoid and up-sampling layer. Finally, the input features were multiplied by α to get the attention map. The contribution of the attention gates was demonstrated using ablation experiments.

Cropped images were downsampled 2, 4, and 8 times, and these downsampled images were input into the network together with the original image to prevent image information loss in the encoder path. A deeply-supervised strategy was applied to force the feature maps in the decoder path to be semantically discriminative and improve model performance (Bin and Karl, 2018).

### Network Description

The source dataset was randomly split into training cohort (80 subjects) and test cohort (20 subjects). Five-fold cross-validation over the training cohort was used for model training and selection. The test cohort was used for the final evaluation of the model. This dataset was used to train the network to segment two regions containing RN and SN–STN, which provided initial weights for the target network. Pre-processed images with a matrix size of 128 × 128 × 3 and corresponding ground truth with a batch size of 32 were fed into the source network to obtain the weights of the model. The training process was stopped when the loss function was no longer reduced for 20 epochs.

**FIGURE 4:**
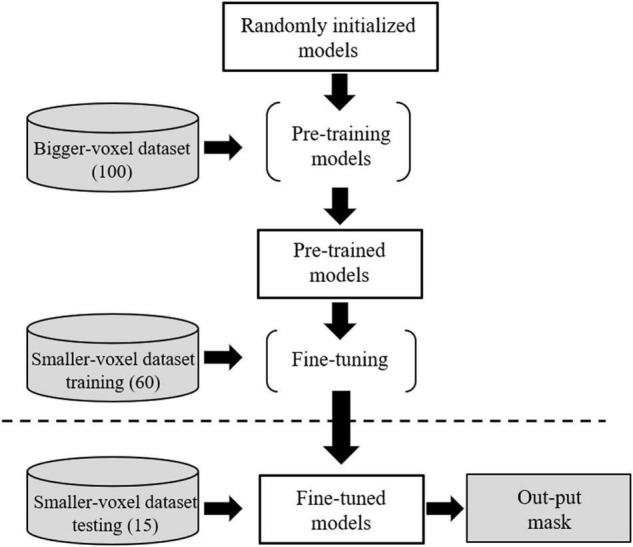
Transfer learning from the source dataset to the target dataset. Voxel sizes were 0.63 × 0.63 × 2.00 and 0.83 × 0.83 × 0.80 mm^3^ for the source and target domain datasets, respectively.

The target dataset was randomly split into training cohort (60 subjects) and test cohort (15 subjects). The pre-trained weights of the source model (excluding the output layers) were used to initialize the transfer learning CNN model (TL-model) and all the transferred weights were fine-tuned to segment RN, SN, and STN during training. The transfer learning procedure is presented in Figure 4.

A non-transfer learning CNN model (NTL-model) was also trained using the target dataset with random weight initialization.

A combination of Dice and cross-entropy loss was used as loss function in the training:


$$D\,i\,c\,e_{l\,o\,s\,s}=1-\frac{2\times \sum_{i=1}^{N}p_{i}\,g_{i}}{\sum_{i=1}^{N}p_{i}+\sum_{i=1}^{N}g_{i}}$$

$$c\,r\,o\,s\,s\,\_\,e\,n\,t\,r\,o\,p\,y=-\frac{1}{N}\,\sum_{i=1}^{N}\left(g_{i}\,\log\left(p_{i}\right)+\left(1-g_{i}\right)\,\log\left(1-p_{i}\right)\right)$$

$$L\,o\,s\,s=D\,i\,c\,e_{l\,o\,s\,s}+c\,r\,o\,s\,s\,\_\,e\,n\,t\,r\,o\,p\,y$$

where the *p*_*i*_ and *g*_*i*_ represent the probability and ground truth of pixel *i*, respectively; *N* is the number of total pixels. Since our models used three output branches to segment three different nuclei, the weighted sums of loss for each branch were used:


$$L\,o\,s\,s_{t\,o\,t\,a\,l}=\lambda_{1}\times L\,o\,s\,s_{1}+\lambda_{2}\times L\,o\,s\,s_{2}+\lambda_{3}\times L\,o\,s\,s_{3}$$

*Loss*_*n*_ (*n* = 1, 2, and 3) represent the loss functions calculated from the three output branches. The weights for the loss function (λ_1_, λ_2_, and λ_3_) were assigned as 0.6, 0.3, and 0.1, respectively. These hyperparameters were optimized by trying different weight values. The Adam algorithm was used to minimize the loss function during back-propagation with an initial learning rate of 10^–4^ (Kingma and Ba, 2015). An early stopping strategy was used to avoid overfitting, the training process was stopped if the loss on the validation dataset was not reduced over 20 epochs.

The models were implemented using Pytorch (version: 1.6.0) and Python (version: 3.7). Experiments were conducted on a workstation equipped with four NVIDIA TITAN XP GPUs. The source codes are available online^3^.

For comparison, an attention U-net model was also trained by using the target dataset and the weights of the Resnet50 model (He et al., 2016) pre-trained on the ImageNet dataset (Russakovsky et al., 2015) were used to initialize the encoder part of the model. During training, all the transferred weights were fine-tuned. All the experiments shared the same environment, hyperparameters, loss function, augmentation strategy and used the same training set and test set.

The performance of all models was evaluated with the corresponding test cohort. Each slice in a case was preprocessed identical to the training data before being fed into the trained U-net to get the predicted 2D probability maps. The results of the five trained models were ensembled by averaging the predicted probability maps to obtain the final segmentation result. A threshold of 0.5 was used to obtain binary segmentation masks before 2D masks of all slices were combined into a 3D volume, in which only the largest connected regions (Bernal et al., 2019) were selected for the final segmentation for each case.

### Statistical Analysis of Segmentation Performance

Dice coefficients were used to assess spatial overlap agreement between manual delineations (ground truth) and automated segmentation. Dice scores were estimated with the following equation:


$$D=\frac{2\times v\,o\,l\,\left(M\,\cap A\right)}{v\,o\,l\,\left(M\right)+v\,o\,l\,\left(A\right)}$$

where M and A represent the manual and automated segmentations, respectively. Meanwhile, vol (⋅) indicates the volume of the segmentation. Segmentation was performed on 2D slices, but the accuracy of deep nuclei segmentation was calculated at the volume level. *D* equals 1 when there is a perfect match between the manual and automated segmentations, and 0 when there is no overlap. The Dice metric was also used to estimate inter-rater segmentation performance to establish a baseline for comparison. A paired *t*-test was used to compare Dice values obtained by the TL and NTL models. A *p*-value of less than 0.05 was deemed significant.

In addition to the Dice coefficients, quantitative values of tissue volume and magnetic susceptibility between the automated and manual segmentations were also compared using Pearson correlation coefficients and Bland-Altman analyses. All statistical analyses were carried out using IBM SPSS Statistics 22.

## Results

**FIGURE 5:**
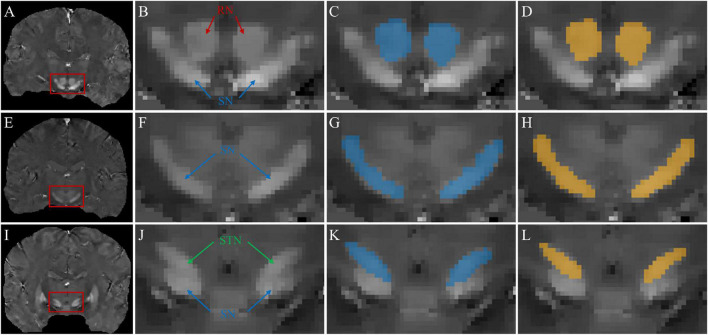
Segmentations of midbrain structures. First column: coronal QSM images **(A,E,I)** showing the RN, SN, and STN (red box). Second column: zoomed-in view **(B,F,J)** of the structures in the red box in the 1st column. Third column: manual segmentations (blue masks) of the RN **(C)**, SN **(G)**, and STN **(K)**. Fourth column: automated segmentations (yellow masks) of the RN **(D)**, SN **(H)**, and STN **(L)**.

Figure 5 shows typical segmentations of the RN, SN, and STN by manual tracing and by the TL-model. Automatic segmentation (Figures 5D,H,L) delineated the nuclei with similar boundaries to manual tracing (Figures 5C,G,K) and captured areas of high susceptibility in the susceptibility maps.

### Dice Similarity Analysis

In the testing cohort of the source dataset, the source model achieved Dice scores of 0.810 ± 0.089 and 0.788 ± 0.067 for the RN and SN-STN, respectively, which indicated that this model could perform preliminary segmentations of the RN and SN-STN.

**FIGURE 6:**
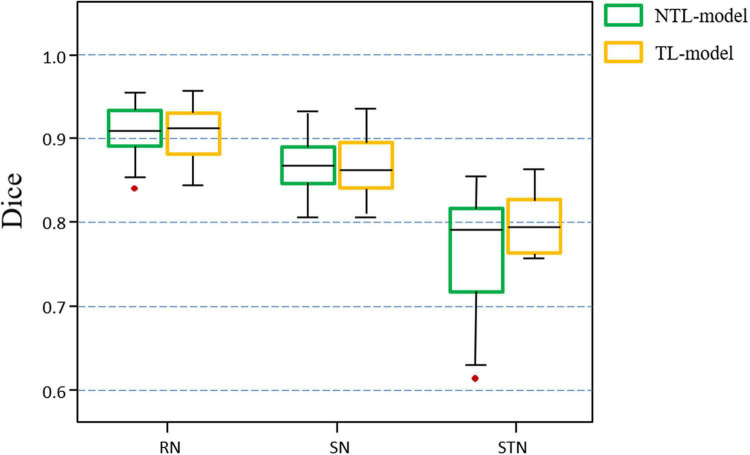
Boxplots of Dice scores achieved by the non-transfer learning CNN model (NTL-model) and the transfer learning CNN model (TL-model) on the test cohort of the target dataset. Colored boxes indicate the 25th–75th percentile range, black bars refer to the median values, and red circles represent outliers (more than 1.5 × the interquartile range away from the box).

Figure 6 shows the distribution of the mean Dice scores for the TL-model and NTL-model on the test cohort of the target dataset. The TL-model achieved Dice scores of 0. 903 ± 0.023, 0.864 ± 0.033, and 0.777 ± 0.066 for the RN, SN, and STN, respectively, while the NTL-model achieved Dice values of 0.903 ± 0.023, 0.866 ± 0.030, and 0.762 ± 0.073 for the RN, SN, and STN, respectively. A paired *t*-test with the Dice values showed that transfer learning improved STN segmentation performance (*p* = 0.07). The median, upper and lower quartiles of the STN Dice scores obtained by the TL-model were higher than those obtained by the NTL-model. According to the box plot, outliers in the segmentation by NTL-model disappeared in the results by the TL-model. Transfer learning reduced training time from 5 h for the NTL-model to 2 h for the TL-model.

The model without attention gate achieved Dice scores of 0.904 ± 0.020, 0.864 ± 0.035, and 0.767 ± 0.079 for the RN, SN, and STN, respectively. The model using the weights of the Resnet50 model pre-trained on the ImageNet dataset achieved Dice scores of 0.903 ± 0.021, 0.861 ± 0.040, and 0.736 ± 0.102 for the RN, SN, and STN, respectively.

For the remainder of this section, all results refer to those of the TL-model.

**FIGURE 7:**
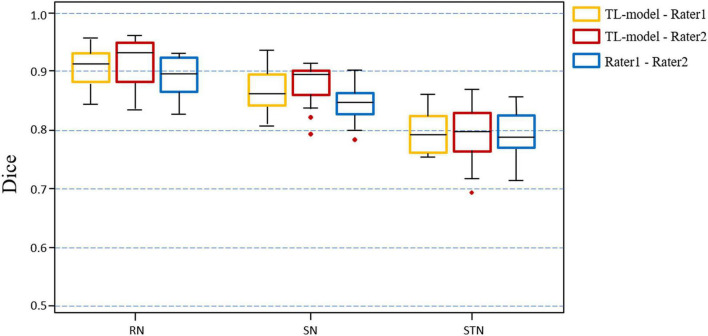
Boxplots illustrating segmentation performance of midbrain gray matter nuclei. Automatic segmentations with transfer learning CNN model (TL-model) are compared to manual segmentations by Rater 1 (yellow boxes) or Rater 2 (red boxes). Dice scores between manual segmentations by two raters (blue boxes) are also presented. Colored boxes indicate the 25th–75th percentile range, black bars correspond to the median value, and red circles represent outliers (more than 1.5 × the interquartile range away from the box).

Figure 7 shows the Dice value distribution using pairwise comparison of the segmentation results from the TL-model and two researchers. Inter-rater Dice scores were 0.891 ± 0.027, 0.845 ± 0.037, and 0.783 ± 0.061 for the RN, SN, and STN, respectively. There were no significant differences between Dice scores from automated segmentation and inter-rater Dice scores in the RN (*t* = 1.391, *p* = 0.186), SN (*t* = 1.883, *p* = 0.081) and STN (*t* = −0.236, *p* = 0.817). Automated segmentation compared with the second rater achieved Dice scores of 0.910 ± 0.028, 0.861 ± 0.031 and 0.772 ± 0.045 for the RN, SN, and STN, respectively.

### Volume and Susceptibility Correlation Analysis

**FIGURE 8:**
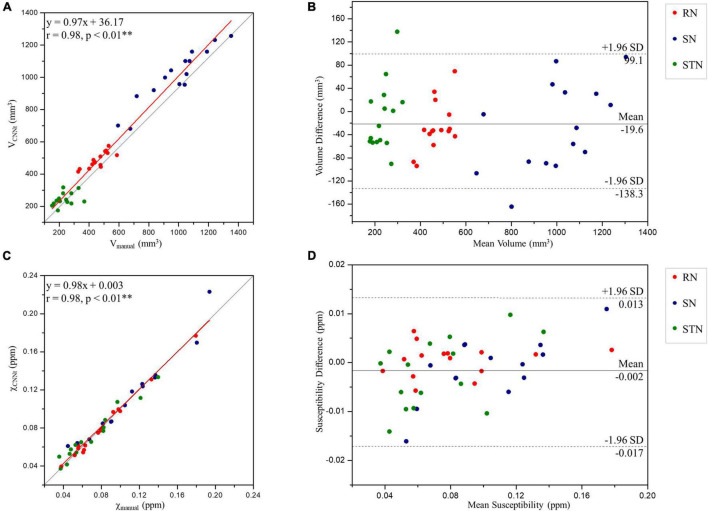
Scatter plots **(A,C)** and Bland-Altman plots **(B,D)** of the volume **(A,B)** and tissue susceptibility values **(C,D)** in the midbrain deep nuclei from the TL-model and manual tracing by rater 1. In the scatter plots, the red and gray lines are the trend line of the linear regression and the line of equality, respectively. In the Bland-Altman plots, the solid and dashed lines indicate the mean difference and 95% confidence level interval, respectively. SD, standard deviation; RN, red nucleus; SN, substantia nigra; STN, subthalamic nucleus. ^**^Indicates significant correlations *p* < 0.01 level.

Region volumes and mean susceptibility values from manual tracing and the TL-model are plotted in Figure 8 and summarized in Table 1. Overall volume and magnetic susceptibility values of the ROIs extracted using the TL-model were significantly correlated (*p* < 0.01) with those obtained using manual delineation, with the overall correlation coefficients r equaling 0.98 (Figures 8A,C). The corresponding susceptibility values from the automated approach showed 95% limits of agreement of −0.002 ± 0.015 ppm with respect to the manual approach (Figure 8D). Volume and susceptibility values of each nucleus extracted with TL-model were also significantly correlated (*p* < 0.01) with those extracted manually (Table 1). All correlation coefficients *r* were larger than 0.8.

**TABLE 1:** Summary of the volumes and mean susceptibility values in the selected regions of interest (ROIs) delineated manually by Rater 1 and the automated convolutional neural network (CNN) method with transfer learning (TL)-model.

ROIs	Volumes (mm^3^)		Correlation	Susceptibility (ppm)		Correlation
	Manual	Automated	*r*	Manual	Automated	*r*
RN	459.0 ± 71.2	485.5 ± 48.1	0.82**	0.082 ± 0.036	0.081 ± 0.036	0.99**
SN	984.5 ± 210.8	1011.1 ± 170.5	0.94**	0.108 ± 0.042	0.111 ± 0.044	0.98**
STN	230.9 ± 63.1	241.1 ± 40.5	0.83**	0.070 ± 0.031	0.072 ± 0.028	0.98**

*RN, red nucleus; SN, substantia nigra; STN, subthalamic nucleus.*

*r: Pearson correlation coefficient.*

**Indicates statistical significance with p < 0.01.

*Volume and susceptibility values are presented as mean ± standard deviation.*

## Discussion

In this work, we segmented the midbrain gray matter nuclei automatically in high-resolution susceptibility maps using a CNN model with transfer learning. The true ellipsoidal shape of the STN was reliably reflected in the high-resolution susceptibility maps, which is the prerequisite for the segmentation of individual deep brain nuclei. The application of the CNN model with transfer learning was another highlight of this study. The combination of these two aspects allowed this automatic segmentation procedure to be performed in single-modal QSM images and yielded comparable results to manual delineation. Dice scores for the RN, SN, and STN were commensurate with inter-rater reliability ratings. Moreover, this proposed segmentation method allowed the volumes and magnetic susceptibility values of midbrain gray nuclei to be reproducibly quantified.

A variety of midbrain structures segmentation studies have been reported (Lim et al., 2013; Li et al., 2016, 2019; Visser et al., 2016; Garzón et al., 2018; Kim et al., 2019; Plassard et al., 2019; Beliveau et al., 2021), which are summarized in Table 2. These studies were mainly based on multi-modality MRI images, including T1-, T2-, T2*-weighted or QSM images. Our mean Dice score between manual and automated segmentation for RN was 0.903, which is comparable with those from previous studies (Visser et al., 2016; Garzon et al., 2018; Li et al., 2019). Further, mean Dice coefficients were 0.864 and 0.777 for SN and STN, respectively, which are higher than those reported in previous studies on segmentation of susceptibility-contrast images (Lim et al., 2013; Visser et al., 2016; Garzon et al., 2018; Zhang et al., 2018; Li et al., 2019), with ranges of 0.65–0.81 and 0.53–0.70 for the SN and STN, respectively.

**TABLE 2:** Summary of state-of-art automatic midbrain nuclei segmentation studies.

Studies	Segmentation methods	Dataset					Dice
		B0	Image type	Voxel size (mm^3^)	No. of subjects	Directly visible STN	
Garzón et al., 2018	Multi-atlas estimation of spatial priors for a Gaussian mixture model	3T	QSM T1WI	0.9 × 0.9 × 1.0 0.9 × 0.9 × 1.0	40 Healthy subjects	Yes	0.88 (RN) 0.81 (SN) 0.66 (STN)
Li et al., 2019	Multi-atlas based on QSM&T1WI	3T	QSM T1WI	1.0 × 1.0 × 1.0 1.0 × 1.0 × 1.2	17 Healthy subjects	No	0.83 (RN) 0.80 (SN) 0.70 (STN)
Plassard et al., 2019	Multi-atlas based on images at 7 T	7T/3T	T1WI_7T_ SWI_7T_ T1WI_3T_	0.7 × 0.7 × 0.7 0.2 × 0.2 × 1.1 1.0 × 1.0 × 1.0	Nine healthy subjects	No	0.65 (SN_left_) 0.70 (STN_left_)
Lim et al., 2013	EvePM atlas created by multimodal imaging data	3T	QSM T1WI DTI	1.2 × 1.2 × 1.2 1.2 × 1.2 × 1.2 2.2 × 2.2 × 2.2	Five healthy subjects	No	0.88–0.90 (RN) 0.86–0.88 (SN/STN)
Visser et al., 2016	Modified MIST method with a Markov random field prior	7T	T2*WI QSM	0.5 × 0.5 × 0.5 0.6 × 0.6 × 0.6	53 Healthy subjects	Yes	0.83–0.88 (RN) 0.68–0.78 (SN) 0.59–0.68 (STN)
Li et al., 2016	Level set method	3T	T2WI	0.5 × 0.5 × 3	10 Patients with PD	No	0.86–0.88 (SN/STN)
Kim et al., 2019	Machine learning, statistical shape and pose relationship learned from 7 T priors	7T/3T	T2WI SWI	0.39 × 0.39 × 1(2) 0.39 × 0.39 × 0.8	80 Subjects	Yes	0.64 (STN)
Beliveau et al., 2021	Five CNN architectures	3T	SWI	0.68 × 0.68 × 2.40	30 Healthy subjects	No	0.83–0.87 (RN) 0.79–0.86 (SN) 0.53–0.66 (STN)
Our study	CNN with transfer learning	3T	QSM	0.83 × 0.83 × 0.8 0.63 × 0.63 × 2.0	175 healthy subjects	Yes	0.90 (RN) 0.86 (SN) 0.78 (STN)

B0, main magnetic field strength; QSM, quantitative susceptibility mapping; T1WI, T1-weighted images; T2WI, T2-weighted images; T2*WI, T2*-weighted images; SWI, susceptibility-weighted images; DTI, diffusion tensor imaging; PD, Parkinson’s disease.

The excellent segmentation achieved in this study can be partly attributed to the high-resolution susceptibility maps (Dimov et al., 2019). The true ellipsoidal shape of the STN is reliably reflected and distinguished from the SN in the high-resolution susceptibility maps, which is the foundation for accurate segmentation of the STN. Inter-rater Dice scores in this study were 0.89, 0.85, and 0.78 for RN, SN, and STN, respectively, which are comparable to those reported in a previous study based on high-resolution 0.5 mm isotropic susceptibility maps from a 7T scanner (Visser et al., 2016), with RN having the highest among the three nuclei reflecting the best conspicuity. Inter-rater Dice score for the STN (0.78) in this study is much higher than those from the previous 3T MRI studies, which ranged from 0.56 to 0.68 (Xiao et al., 2014; Garzon et al., 2018; Zhang et al., 2018; Li et al., 2019). An advantage of QSM is the deconvolution of the local magnetic field, which allows for a better definition of the deep gray matter nuclei better, eliminating blurring caused by magnetic susceptibility artifacts (Li et al., 2012). Previous qualitative and quantitative studies have demonstrated that QSM is highly reproducible and is superior to T2w, T2*w, R2*, and susceptibility-weighted images in the depiction of the STN (Schafer et al., 2012; Liu et al., 2013a). Further, coronal views in high-resolution susceptibility maps allow easier differentiation of the STN from the SN (Schafer et al., 2012), which are very small adjacent structures. However, high-resolution QSM requires increased scan time and leads to a reduced signal-to-noise ratio (Schafer et al., 2012; Liu et al., 2013a). In this study, a voxel size of 0.83 × 0.83 × 0.80 mm^3^ was used to balance scan time and image quality. An advantage of this approximate isotropic voxel size is that the coronal images can be reformatted from the acquired original transverse images, which can help discriminate the STN from SN.

Convolutional neural network model with transfer learning is another attribution for our excellent segmentation performance. Up to now, no study has utilized CNN to segment midbrain structures in high-resolution susceptibility maps, though the CNN model is rapidly evolving in image segmentations (Kim et al., 2019; Beliveau et al., 2021). The size of the high-resolution dataset in our study can hardly meet the requirement of CNN model training. Accordingly, a transfer learning scheme is applied by borrowing initial weights from an existing model used to segment two nuclei on QSM images of larger voxel size, in a manner similar to using a model pre-trained on large-scale natural images in typical transfer learning studies. This application of transfer learning may be more effective because both datasets of brain susceptibility maps contain similar image content in terms of contrasts and structures, except voxel size. The knowledge being “transferred” in the process may involve the basic susceptibility value histogram and the deep gray nuclei geometries. Our results suggest this transfer learning reduces training time and improves the segmentation performance compared with training from scratch, even if the pre-training dataset is small (only 80 cases). This transfer learning may be generalized for modeling new data using weights of the existing CNN model that has been trained on old data, which may have become unavailable and may differ from new data in some characteristics. For example, this transfer learned CNN segmentation may be adapted for longitudinal study of multiple sclerosis lesions (Zhang et al., 2016). Therefore, transfer learning can be used to build models for datasets with similar contrast that are acquired with different scanning parameters or from different MR scanners, when the samples in the newly acquired datasets are not large enough to build a model from scratch.

The proposed method may be used for monitoring brain morphological evolution and iron deposition in normal aging and neurodegenerative diseases. Brain morphology and iron deposition evolve over the entire lifespan (Ward et al., 2014; Caspi et al., 2020), and increased iron deposition in deep gray matter nuclei occurs early in the pathogenesis of several neurodegenerative diseases (Zecca et al., 2004). Our results demonstrated excellent agreement of the region volume and magnetic susceptibility values for each nucleus segmented with the CNN-based method compared to those obtained using manual tracing. Therefore, this automated segmentation procedure may dramatically reduce the amount of manual work and may eliminate operator bias, which will benefit the studies in aging and neurodegenerative diseases.

Accurately automated delineation of the STN can also help clinical treatment for PD patients who are undergoing DBS, because anatomical accuracy of electrode lead placement is critical for a successful surgical outcome (Wodarg et al., 2012). Images obtained from QSM can be imported into existing stereotactic localization software and the automatic segmentation of the STN may improve surgical targeting for DBS lead placement and ultimately result in more efficacious surgery in patients who suffer from advanced PD.

### Limitations

There are several strategies that can further improve the segmentation of midbrain structures. More cases can be included in the training cohort where some cases are synthesized by a generative adversarial network (GAN) (Lan et al., 2020). A 3D model may also make better use of 3D shape and spatial information of the midbrain structures, though training of 3D models also requires more training data. In addition, midbrain structures from each hemisphere may be extracted separately to adapt to specific applications. Using the segmentation from a single rater as the ground truth during training is a limitation of this study, so future work focusing on consensus segmentations from multiple experts will provide ideal ground truth. Future work may also require testing the reliability of automatic segmentation algorithms across imaging devices or institutions and detecting regional volume and susceptibility values not only in healthy subjects with a limited age range, but also in those over a wider age range and/or with neurological disease.

## Conclusion

We have presented an automated segmentation method for the midbrain gray matter nuclei using a combination of high-resolution susceptibility maps and CNN with transfer learning. By using transferred knowledge in a model trained with similar data (acquired with same pulse sequence but different scan parameters) and different labels, a new network for an extended target can be effectively trained with a relatively small data size. This transfer learned CNN allows excellent segmentation of deep gray nuclei on quantitative susceptibility maps. Future studies on brain volumetric change or iron deposition across the lifespan and in neurodegenerative diseases will benefit from this segmentation approach.

## Data Availability Statement

The original contributions presented in the study are included in the article/Supplementary Material, further inquiries can be directed to the corresponding author/s.

## Ethics Statement

The studies involving human participants were reviewed and approved by Human Subject Protection Committee of East China Normal University. The patients/participants provided their written informed consent to participate in this study.

## Author Contributions

JL and GY made substantial contributions to the conception and design of the study. WZ, ZW, and HZ participated in the data acquisition. YidW, WZ, FZ, and YS carried out the study and performed the data analysis. WZ and GL designed and carried out the statistical analysis. WZ performed data interpretation and drafted the manuscript. JL, GY, KG, and YiW revised the manuscript critically for important intellectual content. All authors read and approved the final manuscript.

## Conflict of Interest

YiW owns equity of Medimagemetric LLC, a Cornell spinoff company. The remaining authors declare that the research was conducted in the absence of any commercial or financial relationships that could be construed as a potential conflict of interest.

## Publisher’s Note

All claims expressed in this article are solely those of the authors and do not necessarily represent those of their affiliated organizations, or those of the publisher, the editors and the reviewers. Any product that may be evaluated in this article, or claim that may be made by its manufacturer, is not guaranteed or endorsed by the publisher.

## Footnotes

1
http://pre.weill.cornell.edu/mri/pages/qsm.html

2
http://www.itksnap.org

3
https://github.com/wangyidada/Nuclear_segmentation
