# Supplementary material for: Automated Segmentation of Midbrain Structures in High-Resolution Susceptibility Maps Based on Convolutional Neural Network and Transfer Learning
Source: Front Neurosci. 2022 Feb 10;16:801618. doi: 10.3389/fnins.2022.801618 (PMC8866960; doi:10.3389/fnins.2022.801618)
Supplement: Supplementary file 1 [file Data_Sheet_1.DOCX]

**Material S1.** Estimation on the total number of augmented training samples used in the training processes.

Since each sample was augmented whenever it was used, in a certain sense, the times of sample usage can be regarded as the total number of augmented samples, which can be estimated using the following equation:

$$N=\sum_{i=1}^{5} s\times n\times{epoch}_{i}$$

where *s* indicates the number of slices for each subject, *n* represents the number of subjects used for model training, *i* and *epoch_i_* denote the fold index (5 folds in total) and the number of training epochs for the i-th fold, respectively.

In the source dataset, the number of slices for each subject was 96. There were 80 subjects in the training cohort, 64 of which were used for model training. In the five-fold cross-validation experiments, the numbers of training epochs for each fold were 181, 168, 148, 190, and 212, respectively. So, the total number of augmented training images was about 5.5 million.

In the target dataset, the number of slices for each subject was 192. There were 60 subjects in the training cohort, 48 of which were used for model training. In the five-fold cross-validation experiments, the numbers of training epochs for each fold were 119, 91, 122, 108, and 126, respectively. So, the total number of augmented training images was about 5.2 million.
